# Simultaneous bright- and black-blood three-dimensional whole-heart magnetic resonance imaging for integrated coronary plaque detection and luminal stenosis assessment: A prospective comparison with coronary computed tomography angiography

**DOI:** 10.1016/j.jocmr.2026.102688

**Published:** 2026-01-13

**Authors:** Simon J. Littlewood, Natalie Montarello, Reza Hajhosseiny, Michael G. Crabb, Dongyue Si, Sophie Quick, Anastasia Fotaki, Karl P. Kunze, Ronak Rajani, Claudia Prieto, René M. Botnar

**Affiliations:** a School of Biomedical Engineering and Imaging Sciences, King's College London, London, United Kingdom; b Cardiovascular Directorate, Guy’s and St Thomas’ NHS Foundation Trust, London, United Kingdom; c Department of Cardiology, Chelsea and Westminster Hospital NHS Foundation Trust, London, United Kingdom; d Department of Radiology, Guy’s and St Thomas’ NHS Foundation Trust, London, United Kingdom; e MR Research Collaborations, Siemens Healthcare Limited, Camberley, United Kingdom; f Institute for Biological and Medical Engineering, Pontificia Universidad Católica de Chile, Santiago, Chile; g School of Engineering, Pontificia Universidad Católica de Chile, Santiago, Chile; h Millennium Institute for Intelligent Healthcare Engineering, Santiago, Chile; i Institute for Advanced Study, Technical University of Munich, Garching, Germany

**Keywords:** Coronary plaque, Stable chest pain, Coronary artery disease, CMR

## Abstract

**Background:**

Coronary computed tomographic angiography (CCTA) is a first-line test for anatomical evaluation of the coronary arteries in stable chest pain. Despite technical advances, CCTA requires breath hold and exposes patients to ionizing radiation and contrast agents. Coronary cardiovascular magnetic resonance angiography (CCMRA) has been limited by long and unpredictable scan times, lower spatial resolution, and restricted plaque characterization. We developed a novel cardiovascular magnetic resonance sequence, Bright-blood and black-blood phase SensiTive (BOOST) sequence, which produces a co-registered bright-blood image for lumen visualization and a T1-weighted black-blood image for vessel wall and plaque assessment from a single scan with predictable scan times.

**Objectives:**

To compare the BOOST-CCMRA sequence with CCTA for plaque characterization and stenosis evaluation in patients with stable chest pain.

**Methods:**

Sixty consecutive patients (mean age 56 years, 60% (36/60) male) with stable chest pain were prospectively enrolled. All underwent CCTA followed by BOOST-CCMRA on a 1.5T magnetic resonance imaging scanner. Coronary plaques identified on CCTA were analyzed on the black-blood BOOST image using the signal from plaque to derive the plaque-to-myocardium ratio (PMR); a healthy vessel-to-myocardium ratio (HVMR) was derived as reference. Plaque morphology was assessed by CCTA appearance. Luminal stenosis was assessed on BOOST bright-blood images and compared with CCTA.

**Results:**

Of 60 patients, 35 had plaque on CCTA, with 72 plaques identified. 9 plaques were not detected on BOOST, giving an 88% (63/72) detection success. BOOST showed agreement with CCTA in stenosis grading for 51 of 63 lesions (81%): 23/26 (88%) minimal, 20/24 (83%) mild, 4/7 (57%) moderate, 3/5 (60%) severe, and 1/1 (100%) occlusion. PMR was significantly higher than HVMR (0.64 ± 0.16 vs 0.36 ± 0.11; p < 0.001) across all plaque subtypes (calcified 0.53 ± 0.11, partially calcified 0.70 ± 0.15, non-calcified 0.69 ± 0.16, all p < 0.001 vs HVMR). Hypertension and family history of premature cardiovascular disease were associated with higher PMR values.

**Conclusion:**

The BOOST sequence allows simultaneous evaluation of coronary lumen and plaque characteristics in a single non-contrast CCMRA acquisition, with reliable plaque identification and good agreement with CCTA for stenosis severity assessment. This approach may offer free-breathing alternative, without radiation or contrast, for integrated anatomical and plaque imaging in patients with stable chest pain.

## Graphical abstract

**Figure:**
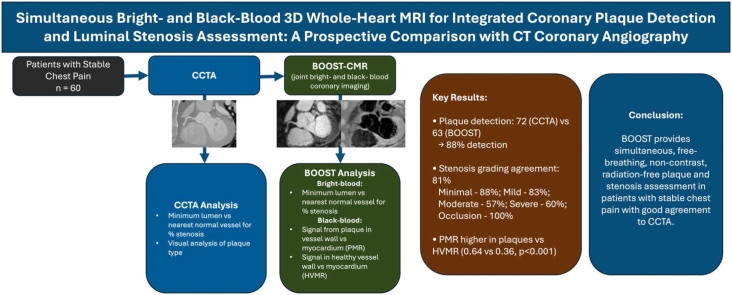

## 1 Introduction

Anatomical imaging has surpassed functional testing of coronary artery disease (CAD). Current guidelines in the United States and Europe indicate coronary computed tomographic angiography (CCTA) as the first-line investigation for patients with stable chest pain at low-to-intermediate risk [1], [2]. Despite this, CCTA is not without its own inherent challenges. It incurs a radiation dose, requires an injection of iodinated contrast media, is limited in its positive predictive accuracy and image quality, and depends heavily on the patient’s ability to sustain an adequate breath hold. At present, there remains a lack of an alternative non-invasive anatomical imaging test to CCTA.

Prior studies have demonstrated the feasibility of cardiovascular magnetic resonance imaging (CMR) for anatomical assessment of the coronary arteries, but these have been limited owing to the reduced spatial resolution of CMR compared to CCTA, long and unpredictable scan times, and lack of comparative plaque morphology assessment.

Coronary plaque can be identified using T1-weighted (T1W) CMR due to the T1 shortening properties of plaque components, such as thrombus, intra-plaque hemorrhage, lipid, and fibroatheroma [3]. The presence of high-intensity plaque (HIP) on T1W CMR is associated with high-risk plaque features seen on near-infrared spectroscopy and an increased risk of future coronary events [4], [5], [6], [7]. To facilitate a more robust and reproducible method for image analysis, Kawasaki et al. [3] were the first to describe the plaque-to-myocardium ratio (PMR) technique, which normalizes T1 plaque signal to adjacent myocardium. This approach mitigates scanner- and sequence-related variability and has enabled the development of reproducible thresholds for identifying high-risk plaque. A PMR (>1.4) has been shown to correlate with high-risk plaque features and predict coronary events [6], [7], [8], [9].

One such technique, which has been successful in utilizing T1W imaging, is the CATCH (coronary atherosclerosis T1W characterization with integrated anatomical reference) [4] sequence, which provides a simultaneous T1W black-blood dataset for plaque visualization with an anatomical reference dataset for coronary visualization. However, this technique suffers from limited contrast between the lumen, coronary wall, and adjacent myocardium, making accurate anatomical assessment of plaque location challenging. CATCH was specifically designed to identify HIP signal and suppress tissue with a lower range of T1 signal, which may have led to an under-identification of total plaque. In addition, the CATCH technique utilized affine respiratory motion correction instead of non-rigid, which may lead to residual motion artifacts. Finally, image reconstruction was performed offline with reconstruction times of approximately 2 h per patient, which is impractical for clinical applications.

In an effort to combat these shortcomings, we proposed the three-dimensional (3D) whole‐heart non-contrast-enhanced Bright‐blood and black‐blOOd phase SensiTive (BOOST) imaging framework [10], [11]. The sequence utilizes a T2-prepared inversion recovery (T2prep-IR) step on the first heartbeat, followed by an unprepared second heartbeat acquisition, which generates two fully co-registered bright- and black-blood volumes (iT2prep-BOOST). The bright-blood image facilitates vessel lumen visualization, whereas the black-blood image is predominantly T1W and used to visualize the vessel wall and plaque. The framework has already been implemented successfully in patients with suspected aortic disease [12] and most recently in patients presenting with suspected non-ST-segment elevation myocardial infarction [9], but never in a typical low-to-intermediate risk stable chest pain population. Previous studies utilizing different vessel wall imaging techniques have sought to identify vulnerable plaque from other plaque types [3], [5], [6], [9] but not to differentiate stable plaque morphologies based on their appearance on computed tomography (CT).

We therefore sought to explore the utility of iT2prep-BOOST CMR imaging in consecutive patients with stable chest pain, against CCTA, for plaque characterization and stenosis evaluation.

## 2 Methods

### 2.1 Study design

This was a prospective single-center study of patients with stable chest pain symptoms who were referred by their clinician for a CCTA to investigate for potential CAD. Patients were approached at the time of their CCTA and offered the opportunity to undergo a research BOOST CMR scan. Informed written consent was obtained from each patient before enrollment. The study was approved by the local institutional review boards and the United Kingdom National Research Ethics Committee (reference number: 18/SC/0441).

### 2.2 Patient population

Patients >18 years of age with typical or atypical chest pain symptoms who were referred for and underwent CCTA were prospectively recruited. Patients were excluded if they did not proceed to contrast angiography after an initial non-contrast calcium score, which was at the discretion of the treating radiologist. Other exclusion criteria included patients with previous coronary artery bypass surgery, intra-coronary stents, pregnancy, breastfeeding, inability to lie flat, body mass index (BMI) >40 kg/m^2^, allergies or contraindications to glycerol trinitrate (GTN), beta-blockers or iodinated contrast agents, severely impaired renal function (estimated glomerular filtration rate <30 mL/min) or any contra-indication to magnetic resonance imaging (MRI) (e.g., implanted cardiac device, cochlear implants, cerebral aneurysm clips, or other as identified on the MRI safety form). Patients with arrhythmias such as atrial fibrillation (AF) and ectopic beats were included; however, those with AF and a ventricular rate >100 beats per minute (bpm) or other significant arrythmias were excluded. Frequent ectopic beats were defined as ≥1 ectopic beat every 10 heartbeats [13]. Patient demographic details were obtained through the hospital electronic patient record system. Variables included age, sex, BMI, ethnicity, and cardiovascular risk factors such as smoking status, hypertension, hypercholesterolemia, diabetes mellitus, and family history of premature cardiovascular disease.

### 2.3 CT coronary angiography

All CCTA examinations were conducted using a third-generation dual-source CT scanner (SOMATOM Force, Siemens Healthineers, Forchheim, Germany), incorporating three-lead electrocardiogram (ECG) synchronization for cardiac gating. Before imaging, patients received 800 μg of sublingual (SL) GTN for coronary vasodilation, and intravenous (IV) metoprolol in 2.5 mg incremental doses was used where necessary to achieve a target heart rate (HR) < 65 bpm. Images were acquired with an isotropic spatial resolution of 0.6 mm using a prospectively ECG-triggered axial scan protocol during an inspiratory breath-hold, covering from the carina to the inferior border of the heart with a gantry rotation time of 250 ms. The scanner automatically determined the ECG pulsing window and padding range, with arrhythmia rejection enabled via the Adaptive Cardio Sequential mode. A triphasic protocol was administered at 6 mL/s, which typically consisted of 70 mL of neat Iohexol (Omnipaque®, GE Healthcare, London, United Kingdom) 370 mg/mL, followed by a 75 mL mixed bolus of contrast: saline (30%:70%) and finally, a 25 mL saline flush. Bolus tracking was employed, with image acquisition triggered once attenuation in the proximal descending aorta exceeded 110 Hounsfield Units. Image reconstruction was performed using ADMIRE (Advanced Modeled Iterative Reconstruction, Siemens Healthineers, Forchheim, Germany), at strength level 2, with a medium-smooth kernel optimized for cardiac imaging.

### 2.4 BOOST sequence design

**Fig. 1:**
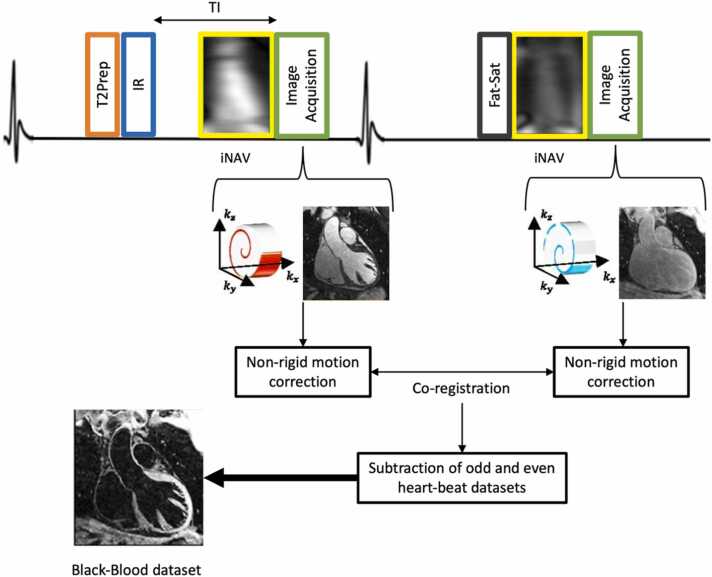
Overview of the iT2prep-BOOST acquisition and reconstruction workflow. The 3D dataset is acquired in an interleaved manner: odd heartbeats apply a T2prep-IR pulse, while even heartbeats apply only a fat saturation pulse. The sequence iNAVs for respiratory motion tracking, enabling 100% respiratory scan efficiency. Respiratory motion estimates derived from iNAVs are used for data binning and the generation of respiratory-phase-resolved 3D images. These images enable estimation of non-rigid 3D motion, which is incorporated into a non-rigid respiratory motion-corrected reconstruction with low-rank patch-based denoising. The resulting co-registered reconstructed images yield bright-blood images for lumen visualization (from odd heartbeats) and black-blood images for vessel wall assessment (via subtraction of odd and even heartbeat reconstructed images). *3D* three-dimensional, *T2prep-IR* combined T2 preparation and inversion recovery, *iNAVs* image-based navigators, *IR* inversion recovery. *BOOST* bright‐blood and black‐blOOd phase SensiTive.

All iT2prep-BOOST acquisitions were performed on a clinical 1.5T MRI scanner (MAGNETOM Sola, Siemens Healthineers, Forchheim, Germany) with a dedicated 32-channel spine coil and 18-channel body coil. iT2prep-BOOST is a free-breathing ECG-triggered 3D balanced steady-state free precession research sequence, in which a T2prep-IR module (T2prep duration = 40 ms; TI = 110 ms) is applied before data acquisition during odd heartbeats, while a fat saturation pulse is applied during even heartbeats (Fig. 1). This results in a bright-blood dataset and a gray-blood reference dataset. The resulting bright-blood and reference datasets undergo magnitude subtraction to generate a co-registered black-blood image. The bright-blood and black-blood images can also be fused to produce a combined vessel lumen and plaque image. The sequence parameters have previously been published [10], [11], [14]. For this study, the following adaptations were used: coronal slab orientation (field of view = 320 × 320 × 112-144 mm^3^), isotropic spatial resolution = 1.2 mm, a three-fold under-sampling factor, flip angle = 90°, TR/TE = 3.35/1.47 ms, and bandwidth = 967 Hz/pixel. Interleaved undersampled *k*-space data were acquired using a variable-density spiral-like Cartesian trajectory with golden-angle rotation [15]. Two-dimensional (2D) iNAVs were incorporated at each heartbeat before image acquisition to enable 100% respiratory efficiency through beat-to-beat respiratory binning and intra-bin translational motion correction [16]. Non-rigid motion-corrected iterative sensitivity encoding reconstruction [17] is performed directly in the scanner software with a reconstruction time of ∼2-3 min. Finally, patch-based low-rank denoising with PROST [18] is performed offline in MATLAB (The MathWorks Inc., Natick, Massachusetts) with a processing time of ∼1-2 min.

### 2.5 BOOST scan protocol

Immediately before scanning, all patients had their HR and blood pressure (BP) recorded to ensure medication safety. Where systolic BP was >100 mmHg, patients were pre-treated with 800 μg of SL GTN to promote coronary vasodilatation. Unless contra-indicated, patients with resting HR >70 bpm were administered IV metoprolol (Betaloc®, AstraZeneca, London, United Kingdom) via a peripheral IV cannula in 2.5 mg increments up to a maximum of 30 mg to achieve a target resting HR of <65 bpm. A three-lead vector ECG was used for cardiac synchronization. A free-breathing four-chamber cine was acquired to set the patient-specific trigger delay and acquisition window, based on the resting period of the right coronary artery (RCA) within the right atrioventricular groove. Where HR was <70 bpm, a mid-diastolic window was favored; where HR was ≥70 bpm or there was significant ectopy, an end-systolic window was chosen.

### 2.6 Study objectives and outcomes

The objective of this study was to evaluate the ability of the iT2prep-BOOST sequence to characterize coronary atherosclerotic plaque compared with CCTA. The primary outcome was the paired difference in PMR between an atherosclerotic plaque and a healthy reference segment within the same coronary artery on iT2prep-BOOST CMR. Secondary outcomes included: (i) comparison of PMR values across different plaque types (calcified, partially calcified, and non-calcified), (ii) agreement between iT2prep-BOOST CMR and CCTA for quantitative luminal stenosis assessment, and (iii) assessment of iT2prep-BOOST image quality, reproducibility, and repeatability.

### 2.7 Coronary data analysis

Both iT2prep-BOOST and CCTA datasets were analyzed using a nine-segment coronary model, which has been described previously [19], [20], [21]. Specifically, the coronaries were divided into left main stem, proximal left anterior descending (LAD), mid-LAD, distal LAD, proximal left circumflex (LCx), distal LCx, proximal RCA, mid-RCA, and distal RCA. This model was chosen to include segments that have the potential to be prognostically significant and to overcome some of the limitations of plaque assessment on CCTA and CMR in small vessels (<2 mm). For each patient, plaques in distinct coronary segments were included for analysis. Where there were multiple plaques in each segment, the plaque causing the most significant stenosis on visual inspection was included for analysis.

#### 2.7.1 CT plaque analysis

CCTA scans were analyzed using specialist software TeraRecon (TeraRecon Inc., Foster City, California) by a Level 3 expert reader in Cardiac CT. Coronary plaques identified by CCTA were categorized as non-calcified, partially calcified, or calcified, based on their visual appearance.

#### 2.7.2 BOOST plaque analysis

Image datasets were reviewed using dedicated CMR imaging software, cvi42 (Circle Cardiovascular Imaging Inc., Calgary, Alberta, Canada). The method to evaluate CMR plaque has previously been described in other studies [3], [4], [6], [7], [9]. In short, using the black-blood dataset, a 2D region of interest (ROI) is delineated at the site of maximal signal intensity in the vessel wall, based on visual assessment (Fig. S2). A second ROI was placed in adjacent myocardium to measure the average myocardial signal, allowing calculation of the PMR [6], [7], [8], [9]. An additional ROI was placed in an area of apparently normal vessel wall to calculate the healthy vessel-to-myocardium ratio (HVMR) from the same coronary vessel as a comparison.

#### 2.7.3 Lumen analysis

Quantitative assessment of luminal stenosis was performed on the CCTA and iT2prep-BOOST bright-blood images by measuring the cross-sectional luminal area at the site of maximum narrowing and comparing it with the nearest area of normal vessel (Fig. S1). This enabled the percentage area stenosis to be calculated. Stenosis severity was classified as minimal (1-24%), mild (25-49%), moderate (50-69%), severe (70-99%), or total occlusion (100%) based on the Coronary Artery Disease–Reporting and Data System (CAD-RADS) 2.0 classification [22].

#### 2.7.4 Image quality, reproducibility, and repeatability

For all iT2prep-BOOST and CCTA scans, qualitative image quality assessment was performed independently based on a 5-point Likert scale: 1, uninterpretable images; 2, poor image quality (limited coronary vessel visibility or noisy image); 3, acceptable image quality (coronary vessel visible but diagnostic confidence low); 4, good image quality (coronary artery adequately visualized and diagnostic quality image); 5, excellent image quality (coronary artery clearly depicted).

To assess repeatability of the results, six patients underwent a second scan with iT2prep-BOOST on the same day, approximately 30 min apart. Before commencing the second scan, patients were brought out of the bore but were not repositioned on the table. To assess inter-reader reproducibility, a second blinded assessor independently drew ROIs and assessed image quality for 10 randomly selected patients.

### 2.8 Sample size and power calculation

The sample size calculation was based on a paired t-test. A clinically meaningful mean difference in PMR of 0.25 with a standard deviation (SD) of paired differences of 0.50 was assumed [9]. We calculated that to achieve 90% power at a two-sided significance level of 0.05, it would require 43 individual plaque lesions. Allowing for a 15% rate of non-diagnostic or excluded segments, the lesion-level requirement increased to 50 lesions. Based on CCTA cohorts, approximately 60% of patients undergoing investigation for stable chest pain are expected to have evidence of CAD [23]. We conservatively estimated an average of 1.5 evaluable lesions per patient with disease, equating to 33 patients with CAD, corresponding to a requirement of approximately 56 patients to be scanned. We therefore opted for a recruitment target of 60 patients.

### 2.9 Statistical analysis

All statistical analyses were performed using IBM SPSS Statistics (version 29.0.2.0; IBM Corp., Armonk, New York) and GraphPad Prism (version 10.5.0, GraphPad Software, San Diego, California). Continuous variables are presented as mean ± SD where normally distributed or median with interquartile range (IQR), otherwise. Categorical variables are reported as counts and percentages. Statistical tests were selected according to the distribution of the data, with parametric methods applied for normally distributed variables and non-parametric methods used otherwise*.* Differences in PMR and HVMR signals were assessed using a paired t-test. PMR values were compared across plaque types (calcified, partially calcified, and non-calcified) using one-way analysis of variance (ANOVA) followed by Tukey’s post-hoc pairwise comparisons with adjustment for multiple testing.

To assess repeatability and inter-reader reproducibility, agreement in PMR values was evaluated using intraclass correlation coefficients (ICCs) (values <0.5 indicative of poor, 0.5-0.75 moderate, 0.75-0.9 good, and >0.90 excellent reliability) [13], and Bland–Altman analysis.

Agreement between CCTA and iT2prep-BOOST for quantitative percentage stenosis assessment was evaluated using Bland–Altman analysis. To assess whether bias varied by plaque type, the Bland–Altman differences (CCTA − iT2prep-BOOST) were compared across three plaque subgroups (calcified, partially calcified, and non-calcified) using the Kruskal–Wallis test. Dunn’s post-hoc multiple comparisons were specified for pairwise comparisons if the overall test was significant.

Relationships between PMR and clinical variables (age, sex, hypertension, hypercholesterolemia, diabetes, smoking history, and family history of premature cardiovascular disease) were assessed using a linear mixed-effects model. A two-sided p-value <0.05 was considered statistically significant.

Finally, image quality scores by the primary reader were rescored by two additional blinded readers in 10 randomly selected cases. Inter-observer agreement was evaluated using ICC analysis.

## 3 Results

A total of 193 patients were screened for inclusion, of whom 71 were excluded for meeting pre-specified exclusion criteria and 62 either declined participation or withdrew their consent before the CMR scan. The remaining 60 patients (36 males) with a mean age of 56 ± 11 years were enrolled, and all successfully completed the CMR scanning with the iT2prep-BOOST sequence (Fig. S3) in a mean scan time of 14.3 ± 2 min (range 10.3-19.2 min). A summary of patient characteristics is presented in Table 1. Forty-eight patients were scanned with mid-diastolic imaging (80%), with the remaining 12 scanned in end-systole (20%) due to HR >70 bpm (n = 5) or arrhythmia such as AF (n = 3) or frequent ventricular ectopics (n = 4). The median image quality score for the CCTA datasets was 4 (IQR: 4-5), indicating good image quality. For iT2prep-BOOST imaging, the median image quality score was 4 (IQR: 3-4.5) (scale 1-5), also indicating overall good image quality across the datasets. There was no significant difference in median image quality between images acquired in mid-diastole and end-systole (4, IQR 3-4 vs 4, IQR 3.75-4.25).

**Table 1:** Patient characteristics.

	Patients n = 60
*Demographics*	
Gender, male	36 (60%)
Age, y	56 [28-78]
Body mass index, kg/m^2^	29 (±3.6)
Hypertension	16 (27%)
Hypercholesterolemia	24 (40%)
Diabetes mellitus	15 (25%)
Family history of premature cardiovascular disease	12 (20%)
Ethnicity	
White	40 (67%)
Black	4 (7%)
Asian	11 (18%)
Other	5 (8%)
Smoking	
Current smoker	6 (10%)
Former smoker	11 (18%)
Non-smoker	43 (72%)
*Physiology*	
Heart rate, bpm	64 (±8)
Sinus rhythm	53 (88%)
Atrial fibrillation	3 (5%)
Frequent ectopic beats	4 (7%)
Systolic blood pressure, mmHg	132 (±16)
*Pre-medication*	
Glycerol trinitrate (800 μg)	58 (97%)
Intravenous metoprolol	41 (68%)
Dose, mg	10 (±6)

Summary of baseline patient characteristics (n = 60), including demographics (sex, age, BMI, cardiovascular risk factors, ethnicity and smoking status), physiological measures (heart rate, rhythm and systolic blood pressure), and use of pre-medication. Categorical variables are presented as counts, n (%). Continuous variables are presented as mean (SD), except age, which is presented as median [range].

### 3.1 Coronary plaque analysis

**Fig. 2:**
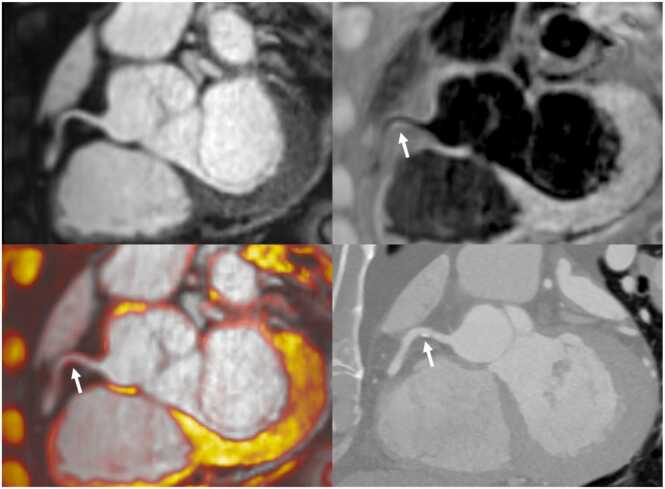
Plaque visualization in the proximal RCA using iT2prep-BOOST and CCTA. Top left: BOOST bright-blood image; top right: BOOST black-blood image; bottom left: fusion image of bright- and black-blood BOOST; bottom right: corresponding CCTA image. White arrows indicate the location of an atherosclerotic plaque in the proximal RCA, which appears hyperintense on the black-blood image and corresponds to a partially calcified lesion on CCTA. *RCA* right coronary artery, *CCTA* coronary computed tomography angiography, *BOOST* bright‐blood and black‐blOOd phase SensiTive

**Fig. 3:**
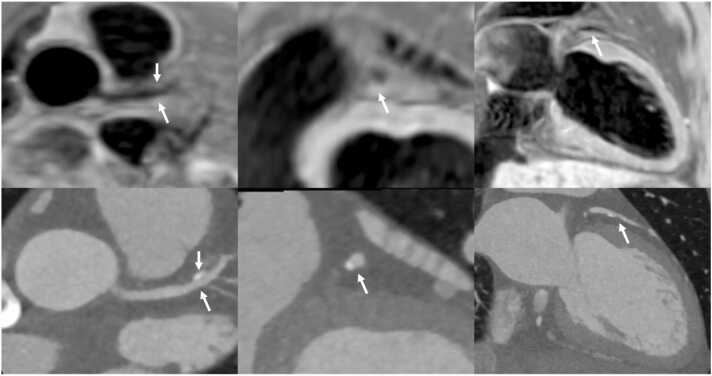
Comparison of iT2prep-BOOST black-blood imaging and CCTA in a patient with partially calcified plaque in the LAD artery. The top row demonstrates black-blood BOOST images in different views, highlighting the hyperintense signal from the partially calcified plaque in the LAD (white arrows). The bottom row shows the corresponding CCTA views of the same vessel. *LAD* left anterior descending, *BOOST* bright‐blood and black‐blOOd phase SensiTive, *CCTA* coronary computed tomography angiography

**Fig. 4:**
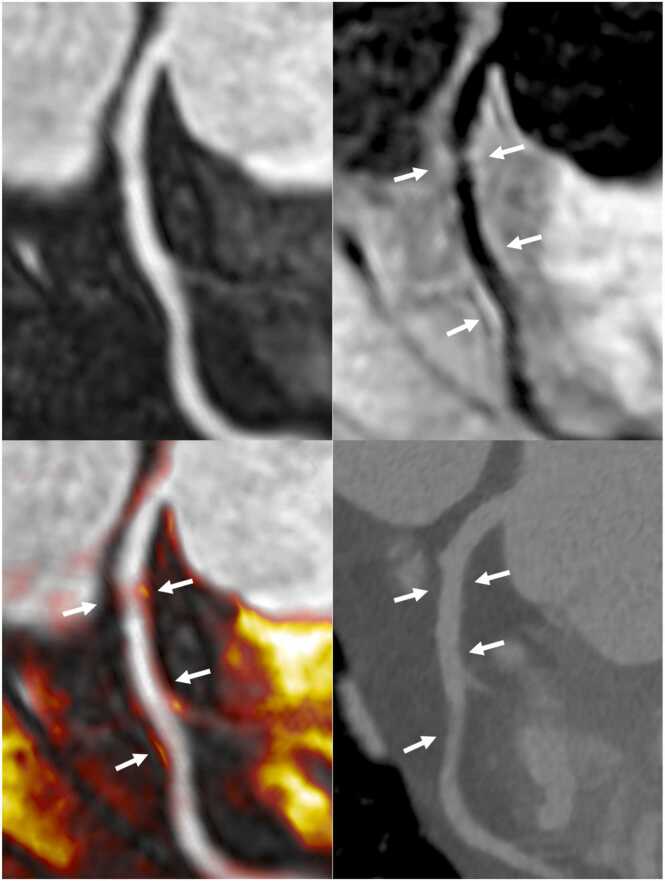
Multiplanar reformats of iT2prep-BOOST and CCTA imaging of a patient with CAD in the proximal and mid-LAD artery. Top left: BOOST bright-blood image demonstrating the coronary lumen; top right: BOOST black-blood image demonstrating the vessel wall; bottom left: fusion image of bright- and black-blood BOOST; bottom right: corresponding CCTA image. White arrows indicate the location of atherosclerotic plaques in the proximal and mid-LAD, which appear hyperintense on the black-blood image and correspond to non-calcified lesions seen on CCTA. *CCTA* coronary computed tomography angiography, *CAD* coronary artery disease, *LAD* left anterior descending, *BOOST* bright‐blood and black‐blOOd phase SensiTive

**Fig. 5:**
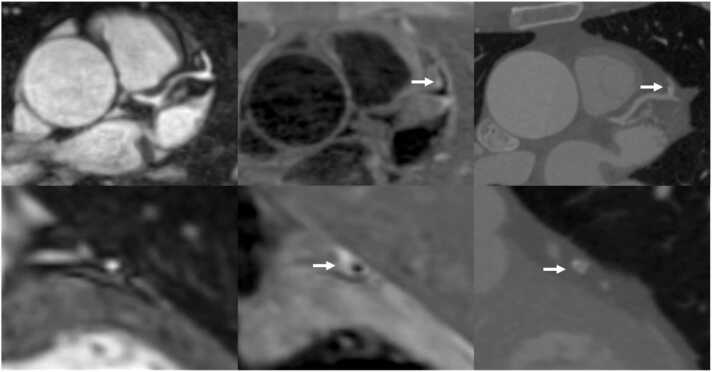
iT2prep-BOOST and CCTA assessment of a mid-LAD plaque. Top row: axial iT2prep-BOOST bright-blood image (left), black-blood image (center), and corresponding CCTA (right). Bottom row: cross-sectional views at the level of a plaque in the mid-LAD using the same modalities. White arrows indicate the location of the atherosclerotic plaque, which appears hyperintense on the black-blood image and corresponds to a partially calcified lesion on CCTA. *CCTA* coronary computed tomography angiography, *LAD* left anterior descending, *BOOST* bright‐blood and black‐blOOd phase SensiTive

**Fig. 6:**
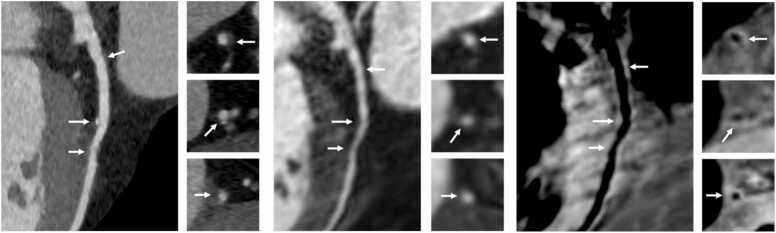
Multiplanar reformat and cross-sectional views of the LAD artery using CCTA and iT2prep-BOOST imaging. Left: CCTA demonstrating multiple plaques (white arrows) in the LAD. Middle: iT2prep-BOOST bright-blood images of the same segments. Right: iT2prep-BOOST black-blood images showing corresponding plaques (white arrows). *LAD* left anterior descending, *CCTA* coronary computed tomography angiography, *BOOST* bright‐blood and black‐blOOd phase SensiTive

**Fig. 7:**
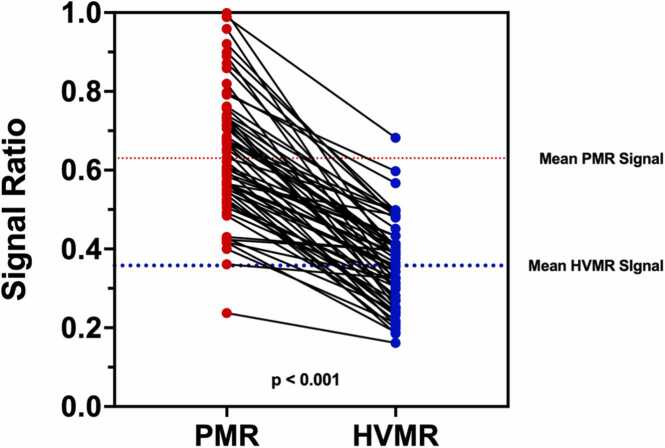
Paired comparison of PMR and healthy HVMR on iT2prep-BOOST. Each red dot represents a single coronary plaque, with lines connecting paired PMR and HVMR values from the same patient. PMR values were significantly higher than HVMR values (mean difference 0.28, 95% CI 0.25-0.32, p < 0.001), indicating increased signal in plaque regions relative to healthy vessel segments. *PMR* plaque-to-myocardium ratio, *HVMR* healthy vessel-to-myocardium ratio, *BOOST* bright‐blood and black‐blOOd phase SensiTive, *CI* confidence interval

Out of 60 patients scanned, 35 patients had CAD identified by CCTA and were included in the plaque analysis. Seventy-two plaques in distinct coronary segments were identified, making an average of two plaques per patient. Nine plaques were not detected on the corresponding iT2prep-BOOST, leaving a total of 63 plaques available for analysis, corresponding to an 88% (63/72) detection success rate for the iT2prep-BOOST compared to CCTA. Representative cases are demonstrated in Fig. 2, Fig. 3, Fig. 4, Fig. 5, Fig. 6. A paired t-test showed the mean PMR (0.64 ± 0.16) was significantly higher than the mean HVMR (0.36 ± 0.11), with a mean difference of 0.28 (95% confidence interval [CI]: 0.25-0.32, p < 0.001) (Fig. 7).

**Fig. 8:**
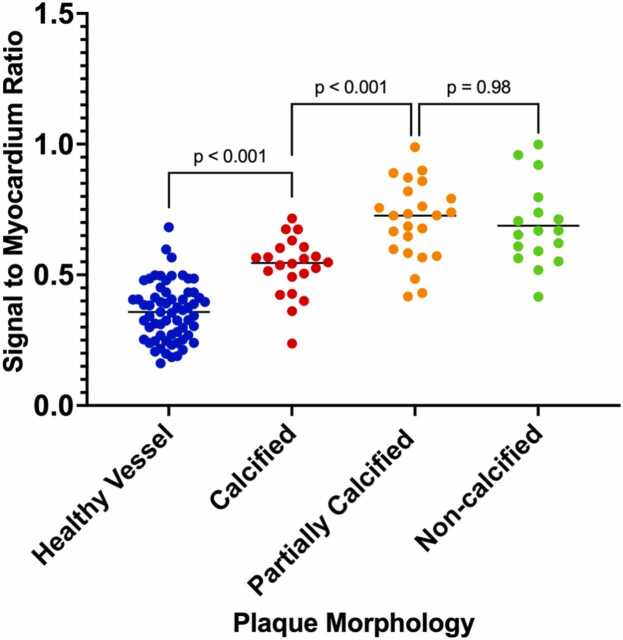
Subgroup analysis of PMR across plaque morphologies and healthy vessels. Individual PMR values are shown for healthy vessels (blue), calcified plaques (red), partially calcified plaques (orange), and non-calcified plaques (green). A one-way ANOVA demonstrated a significant difference among groups (p < 0.001). Tukey’s post-hoc test showed significantly higher PMR higher in all plaque types compared to healthy vessels (healthy vs calcified: mean difference −0.17, 95% CI −0.254 to −0.09, p < 0.001; healthy vs partially calcified: −0.35, 95% CI −0.42 to −0.27, p < 0.001; healthy vs non-calcified: −0.33, 95% CI −0.42 to −0.24, p < 0.001). Both partially calcified and non-calcified plaques showed significantly higher PMR than calcified plaques (mean difference −0.17, 95% CI −0.27 to −0.08, p < 0.001 and mean difference −0.16, 95% CI −0.26 to −0.05, p < 0.001, respectively). No significant difference was observed between partially calcified and non-calcified plaques (mean difference 0.02, 95% CI −0.09 to 0.12, p = 0.98). *PMR* plaque-to-myocardium ratio, *ANOVA* analysis of variance, *CI* confidence interval

Subgroup analysis by plaque type as identified by CCTA is presented in Fig. 8. PMR was significantly higher in all plaque types (calcified, n = 22; partially calcified, n = 24; and non-calcified, n = 17) compared to healthy vessels (healthy vs calcified: mean difference −0.17, 95% CI −0.254 to −0.09, p < 0.001; healthy vs partially calcified: −0.35, 95% CI −0.42 to −0.27, p < 0.001; healthy vs non-calcified: −0.33, 95% CI −0.42 to −0.24, p < 0.001). Furthermore, both partially calcified and non-calcified plaques showed significantly higher PMR than calcified plaques (mean difference −0.17, 95% CI −0.27 to −0.08, p < 0.001 and mean difference −0.16, 95% CI −0.26 to −0.05, p < 0.001, respectively). No significant difference was observed between partially calcified and non-calcified plaques (mean difference 0.02, 95% CI −0.09 to 0.12, p = 0.98).

Results from a linear regression analysis to evaluate the association between patient cardiovascular risk factors and PMR are presented in Table 2. A statistically significant association was observed with hypertension (β = 0.074, 95% CI: 0.003-0.145, p = 0.050) and family history of premature cardiovascular disease (β = 0.092, 95% CI: 0.018-0.166, p = 0.025), indicating higher PMR values in these subgroups. Other variables, including age, sex, cholesterol, diabetes, and smoking status, were not significantly associated with PMR (all p > 0.05).

**Table 2:** Association between cardiovascular risk factors and PMR.

Variable	Estimate (β)	Standard Error	F	95% CI	p-value
Sex (M)	0.042	0.051	0.684	−0.058 to 0.142	0.412
Age	−0.001	0.002	0.171	−0.005 to 0.003	0.681
HTN	**0.074**	**0.036**	**4.306**	**0.003 to 0.145**	**0.05**
Cholesterol	0.037	0.042	0.771	−0.045 to 0.119	0.383
Diabetes	−0.030	0.045	0.441	−0.118 to 0.058	0.510
Smoker	−0.021	0.031	0.454	−0.082 to 0.040	0.509
FHx of CVD	**0.092**	**0.038**	**5.772**	**0.018 to 0.166**	**<0.05**

*PMR* plaque-to-myocardium ratio*, M* male*, HTN* hypertension*, CI* confidence interval*, FHx* family history*, CVD* cardiovascular disease.

Results from linear regression analysis demonstrating the relationship between each patient-level variable and PMR. Estimates (β) reflect the adjusted mean difference in PMR per unit or category. F denotes the regression test statistic (ratio of variance explained to residual error). 95% CI and p-values are shown for each effect. Significant associations are highlighted in bold for HTN and FHx of premature CVD

### 3.2 Stenosis severity

**Fig. 9:**
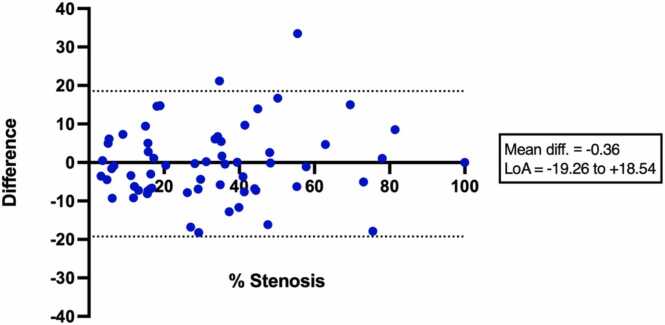
Bland–Altman analysis comparing percentage stenosis assessed by CCTA and iT2prep-BOOST. The plot shows the difference (CCTA − iT2prep-BOOST) against the average stenosis of the two techniques. The solid line represents the mean bias (−0.36%), indicating a negligible difference between the two techniques. The dashed lines represent the 95% LoA (−19.3% to 18.5%), reflecting significant variability between methods for individual lesions. Positive values indicate that CCTA measured a greater stenosis compared with iT2prep-BOOST. *CCTA* coronary computed tomography angiography, *BOOST* bright‐blood and black‐blOOd phase SensiTive

Based on the CAD-RADS 2.0 classification, iT2prep-BOOST demonstrated agreement with CCTA in stenosis severity grading for 51 of 63 lesions (81%). By category, agreement was 26 vs 23 (88%) for minimal, 24 vs 20 (83%) for mild, 7 vs 4 (57%) for moderate, 5 vs 3 (60%) for severe, and 1 vs 1 (100%) for total occlusion (Table 3). Beyond categorical agreement, direct comparison of percentage stenosis was performed to assess potential bias between modalities. Bland–Altman analysis comparing CCTA and iT2prep-BOOST for calculated percentage stenosis demonstrated a negligible mean bias of −0.36% ± 9.64 (95% limits of agreement (LoA) −19.3% to 18.5%) (Fig. 9). Negative differences indicate that iT2prep-BOOST reported greater stenosis values than CCTA. A subgroup analysis assessed the difference in percentage stenosis across three plaque subtypes (calcified, partially calcified, and non-calcified). Median bias (CCTA − iT2prep-BOOST) was 0.95% (IQR: −7.6% to 8.4%) for calcified plaques (n = 22), 0.87% (IQR: −6.9% to 9.1%) for partially calcified plaques (n = 24), and 1.02% (IQR: −8.1% to 7.8%) for non-calcified plaques (n = 17). There was no statistically significant difference in stenosis bias among plaque subtypes (Kruskal–Wallis test = 0.026, p = 0.99) (Fig. S4).

**Table 3:** Comparison of CAD-RADS stenosis severity grading: CCTA vs iT2prep-BOOST.

CAD-RADS category	CCTA lesions (n)	iT2prep-BOOST agreement (n)	Agreement (%)
Minimal	26	23	88%
Mild	24	20	83%
Moderate	7	4	57%
Severe	5	3	60%
Occluded	1	1	100%
Total	63	51	81%

*CAD-RADS* Coronary Artery Disease–Reporting and Data System, *CCTA* coronary computed tomography angiography, *BOOST* bright‐blood and black‐blOOd phase SensiTive

CAD-RADS stenosis severity categories assigned by coronary computed tomography angiography (CCTA) versus iT2prep-BOOST, reporting the number of lesions per category, the number classified in agreement by iT2prep-BOOST, and the corresponding agreement percentage.

### 3.3 Repeatability and reproducibility

Intra- and inter-reader analysis of PMR was undertaken. Six patients underwent repeat scanning with the iT2prep-BOOST. Four patients had plaque identified by CCTA, with a total of seven lesions identified. ICC analysis using a two-way mixed-effects model showed good reliability for individual lesion PMR measurements (ICC = 0.78, 95% CI: 0.16-0.96, p = 0.011), and excellent reliability for averaged measures across all patients (ICC = 0.88).

Inter-observer agreement was assessed in ten randomly selected patients who were independently evaluated by two blinded reviewers. ICC analysis using a two-way random-effects model with absolute agreement definition demonstrated excellent reliability for PMR measurements (ICC = 0.874, 95% CI: 0.569-0.967, p < 0.001). The corresponding average-measures ICC was 0.933, indicating strong agreement when values from both readers are averaged. Bland–Altman analysis was also performed. The mean difference between the two reviewers was −0.007, indicating minimal systematic bias. The 95% LoA ranged from −0.182 to +0.169, with all data points falling within these bounds (Fig. S5).

Inter-reader agreement for image quality among the three readers in ten randomly selected cases was good, with a two-way random-effects, absolute-agreement ICC of 0.65 (95% CI 0.29-0.89) for single measurements and 0.85 (95% CI 0.55-0.96) for the mean of the three readers (p < 0.001 for both).

## 4 Discussion

Current international guidelines recommend CCTA as the first-line investigation for patients with stable chest pain, reflecting its high diagnostic accuracy, widespread availability, and established prognostic value [1], [2], [24]. CCTA provides reliable luminal assessment, but its limitations are well recognized. These include exposure to ionizing radiation and iodinated contrast, reduced specificity in heavily calcified arteries due to blooming artifact and reliance on the patient’s ability to sustain an adequate breath hold. Recent technical advances in CCTA, notably with photon-counting detector CT, offer improved spatial resolution, reduced blooming artifacts from calcified plaques, and enhanced plaque compositional assessment compared with conventional multi-detector CT [25], [26], [27]. However, these systems remain limited in availability and continue to rely on ionizing radiation, iodinated contrast, and the patient’s ability to sustain breath hold. Moreover, accurate identification of plaque phenotypes remains clinically relevant, as high-risk features predict future coronary events independent of stenosis severity [28], [29]. While CCTA can reliably detect calcified plaque and has shown potential for high-risk plaque detection, alternative non-invasive techniques capable of providing both anatomical and plaque compositional information are of considerable interest.

CMR offers the potential for a comprehensive assessment of CAD using free-breathing techniques and without ionizing radiation or contrast administration. Early feasibility studies demonstrated that T1W CMR could detect atherosclerotic plaque based on differences in intrinsic tissue relaxation properties [3], [6]. However, these approaches required separate scans to generate a bright-blood image for coronary anatomy and a T1W image for plaque visualization, which results in long scan times and with potential for mis-registration between lumen and plaque scans, which has hindered accurate plaque localization and reproducibility. The CATCH sequence represented an advance by enabling simultaneous T1W and anatomical datasets, with HIP signals shown to correlate with high-risk features on near-infrared spectroscopy [4], [5], [7], [30]. However, as CATCH was optimized to accentuate HIPs and suppress plaque with long T1 relaxation time, it may under-detect plaques with inherently lower signal intensity, such as calcified lesions.

Recent technical developments have addressed many of these shortcomings. The BOOST framework integrates a free-breathing acquisition with image-based navigation, 3D non-rigid motion correction, and simultaneous, fully co-registered bright- and black-blood images [11], [31]. This enables 100% respiratory scan efficiency, predictable scan times, and improved spatial resolution compared to earlier methods. Importantly, in-line reconstruction reduces post-processing times, enhancing clinical practicality. iT2prep-BOOST has been evaluated in acute coronary syndromes, demonstrating feasibility for plaque detection and vessel visualization using invasive coronary angiography with intravascular imaging as the reference standard [9]. However, its diagnostic performance in stable chest pain populations, where CCTA is the standard of care, has not previously been systematically studied.

In this study, we compared the diagnostic ability of iT2prep-BOOST CMR to CCTA in the evaluation of plaque identification, morphology, and stenosis severity. A key finding is the robust capability of the iT2prep-BOOST sequence to identify coronary plaques, with an overall detection rate of 88% relative to CCTA. Importantly, plaques detected by iT2prep-BOOST demonstrated a significantly higher PMR than healthy vessel walls, with a mean difference of 0.28 (p < 0.001). This confirms the technique's ability to differentiate atherosclerotic plaque from healthy wall tissue. Additionally, subgroup analysis revealed that PMR values were significantly higher in both partially calcified and non-calcified plaques compared to calcified plaques. Interestingly, no significant difference was observed between the PMR of partially calcified and non-calcified plaques. Furthermore, calcified plaques had a significantly higher PMR than healthy vessel walls, which might not have been expected, as calcium typically produces a low signal on MRI [32]. One such explanation may be that these plaques contain some non-calcified material which is not appreciated on CT due to the blooming artifact from calcium, or, the surface relaxation phenomenon, where particulate calcium can restrict the motion of water molecules nearer its surface, effectively shortening the T1 relaxation time, causing a high signal on T1W images [33], [34]. Plaques that were unsuccessfully detected by the iT2prep-BOOST technique were universally very small and calcified. As the spatial resolution of the iT2prep-BOOST (1.2 mm) sequence used in this study was approximately half that of the CCTA (0.6 mm), it is likely these small plaques fell below the detectable resolution.

The bright-blood datasets allowed for lumen visualization and quantitative assessment of stenosis severity. Based on the CAD-RADS 2.0 classification, 81% of lesions detected by iT2prep-BOOST were in the same stenosis severity class as the corresponding CCTA. Bland–Altman analysis demonstrated an overall negligible bias between iT2prep-BOOST and CCTA, with the majority of data points falling within the 95% LoA. However, the wide 95% LoA (−19.3% to 18.5%) suggest considerable variability for individual lesion measurements, highlighting the need for further refinement. This variability did not differ significantly across plaque subtypes (calcified, partially calcified, or non-calcified). These findings offer cautious optimism for the iT2prep-BOOST technique as an alternative for anatomical stenosis assessment in patients with suspected CAD. Greater variability seen in moderate and severe lesions likely reflects the low numbers in these groups, differences in spatial resolution, and effects of calcium blooming, leading to potential overestimation of stenosis on CCTA [35]. However, there were not enough large, calcified lesions causing severe stenosis in this study to draw any firm conclusions.

Regression analysis indicated that hypertension and family history of CAD were associated with higher PMR values, whereas other conventional risk factors such as age, sex, cholesterol, diabetes, and smoking did not show significant associations. This may reflect either limited statistical power or the multifactorial nature of plaque composition that is not fully explained by traditional risk factors. These observations suggest that certain risk profiles may correspond to plaques with higher signal intensity.

Repeatability and inter-observer reproducibility for PMR were good to excellent, with ICC values exceeding 0.78 for repeated scans and 0.87 for independent observers. However, CIs were broad, reflective of the low sample sizes in these analyses. These findings support the stability of PMR measurements when scans are repeated under the same conditions and suggest excellent agreement between independent readers, supporting the reproducibility of PMR assessment.

### 4.1 Clinical implications

The ability to simultaneously assess luminal stenosis and plaque characteristics using a single non-contrast-enhanced MRI sequence has important clinical implications. It could potentially reduce reliance on ionizing radiation and iodinated contrast media, offering an alternative for patients who may be sensitive to these factors, such as younger females, pregnant patients, and those with contrast allergy or renal impairment. Crucially, iT2prep-BOOST offers a free-breathing scan, which cannot be achieved with CT. Furthermore, HIPs are associated with features of vulnerable plaque and an increased risk of subsequent coronary events [6], providing information that is not readily available from attenuation-based plaque assessment on CCTA. This additional information may help to refine risk stratification and guide targeted preventive therapies. There is also the potential to combine lumen and plaque assessment with tissue characterization and stress perfusion CMR to provide a comprehensive cardiac assessment in a single study, reducing patient visits and improving efficient use of resources. However, the long scan times for iT2prep-BOOST CMR (∼14 min) compared to CT (5-10 s) are a significant limiting factor when considering efficient clinical workflows and resource allocation. Finally, the disparity in scanner availability and cost between CT and CMR must be acknowledged. In the UK, cardiac CT is available in the majority of acute care hospitals, whereas CMR is largely restricted to specialist referral centers [36], which substantially limits the accessibility of the BOOST technique to larger patient populations.

## 5 Limitations

Several limitations should be acknowledged. First, this was a single-center study performed on a single MRI scanner and with a modest sample size, limiting generalizability and statistical power for subgroup analyses. In particular, there were unexpectedly low numbers of moderate and severe stenotic lesions. While PMR values were stable on repeat scanning, such tightly controlled conditions are uncommon in routine clinical practice, and PMR is likely to be influenced by scanner vendor, field strength, and specific sequence parameters. As a result, the reproducibility estimates reported here may not directly translate across platforms. Second, non-White ethnic groups were under-represented, limiting the generalizability of the findings in these groups. Third, CCTA served as the reference standard for plaque detection and stenosis grading. Although CCTA is widely accepted as the non-invasive gold standard, invasive coronary angiography with intravascular imaging is considered the gold standard technique for assessing plaque composition [37], [38]. Fourth, PMR was measured per plaque, while risk factors were assessed at the patient level, which may not fully capture the biological complexity of plaque behavior. Fifth, the study design excluded patients who did not proceed to contrast-enhanced CCTA following a high initial calcium score. This was to facilitate a fair comparison of luminal assessment. However, this would be an interesting group of patients to investigate with the iT2prep-BOOST technique, as CCTA struggles with lumen assessment in heavily calcified vessels due to blooming artifact. Sixth, while the iT2prep-BOOST sequence demonstrated robust image quality overall, artifacts related to arrhythmia or residual respiratory motion were observed in some cases. Finally, the lack of patient outcome data available prevents assessment of the prognostic value of the iT2prep-BOOST technique and its potential impact on clinical decision-making.

## 6 Conclusions and Future Directions

To our knowledge, this study is the first to demonstrate the utility of the iT2prep-BOOST CMR sequence in the detection of coronary atherosclerotic plaque and luminal stenosis in a typical low-to-medium risk population with stable chest pain symptoms. Future efforts should focus on techniques to reduce the scan time while maintaining or improving the spatial resolution. Efforts should be made to incorporate the sequence at other field strengths and scanner vendors with harmonized acquisition protocols and phantom-based calibration to enable cross-platform scaling of PMR values. Incorporation of automated segmentation and machine learning-based analysis may streamline workflow and enhance reproducibility. The clinical significance of elevated PMR requires longitudinal studies to determine whether these signal characteristics predict future coronary events. Large multi-center studies are now warranted to explore the possibility of integration of iT2prep-BOOST into routine clinical pathways as an alternative non-invasive technique or adjunct to CCTA in selected patient populations.

## Funding

The authors acknowledge financial support from (1) King’s BHF Centre for Award Excellence RE/24/130035, BHF PG/18/59/33955, RG/20/1/34802, and FS/CRTF/20/24011; (2) 10.13039/501100000266EPSRC EP/V044087/1, EP/P001009/1, EP/P032311/1, EP/P007619; (3) Wellcome EPSRC Centre for Medical Engineering (NS/A000049/1); (4) Millennium Institute for Intelligent Healthcare Engineering ICN2021_004, Fondecyt 1250261, Fondecyt 1250252; (5) IMPACT, Center of Interventional Medicine for Precision and Advanced Cellular Therapy, Santiago, Chile. ANID—Basal funding for Scientific and Technological Center of Excellence, IMPACT, #FB210024; (6) the Department of Health through the 10.13039/501100000272National Institute for Health Research (NIHR) comprehensive 10.13039/100014461Biomedical Research Centre award; (7) NIHR Cardiovascular MedTech Co-operative and (8) the Technical University of Munich – Institute for Advanced Study. The views expressed are those of the authors and not necessarily those of the BHF, NHS, the NIHR, or the Department of Health.

## Author contributions

**Ronak Rajani:** Writing – review & editing, Methodology, Investigation, Conceptualization. **Karl P. Kunze:** Writing – review & editing, Software. **Anastasia Fotaki:** Writing – review & editing. **Sophie Quick:** Writing – review & editing, Investigation. **Natalie Montarello:** Writing – review & editing, Validation, Investigation, Formal analysis. **Simon J. Littlewood:** Writing – review & editing, Writing – original draft, Visualization, Validation, Resources, Project administration, Methodology, Investigation, Formal analysis, Data curation. **Ren**é **M. Botnar:** Writing – review & editing, Visualization, Supervision, Methodology, Funding acquisition, Conceptualization. **Claudia Prieto:** Writing – review & editing, Conceptualization. **Dongyue Si:** Writing – review & editing. **Michael G. Crabb:** Writing – review & editing. **Reza Hajhosseiny:** Writing – review & editing.

## Declaration of competing interests

The authors declare the following financial interests/personal relationships which may be considered as potential competing interests: Karl P. Kunze reports a relationship with Siemens Healthcare Limited that includes employment. The other authors declare that they have no known competing financial interests or personal relationships that could have appeared to influence the work reported in this paper.

## Data Availability

The data underlying this article will be shared on reasonable request to the corresponding author.

## References

[1] Vrints C., Andreotti F., Koskinas K.C., Rossello X., Adamo M., Ainslie J. (2024). 2024 ESC Guidelines for the management of chronic coronary syndromes: developed by the task force for the management of chronic coronary syndromes of the European Society of Cardiology (ESC) Endorsed by the European Association for Cardio-Thoracic Surgery (EACTS). Eur Heart J.

[2] Gulati M., Levy P.D., Mukherjee D., Amsterdam E., Bhatt D.L., Birtcher K.K. (2021). 2021 AHA/ACC/ASE/CHEST/SAEM/SCCT/SCMR Guideline for the evaluation and diagnosis of chest pain: a report of the American College of Cardiology/American Heart Association Joint Committee on clinical practice guidelines. Circulation.

[3] Kawasaki T., Koga S., Koga N., Noguchi T., Tanaka H., Koga H. (2009). Characterization of hyperintense plaque with noncontrast T1-weighted cardiac magnetic resonance coronary plaque imaging: comparison with multislice computed tomography and intravascular ultrasound. JACC Cardiovasc Imaging.

[4] Xie Y., Pang J., Kim Y.J., Yang Q., Kim J.-S., Nguyen C.T. (2016). Coronary atherosclerosis T1-weighed characterization with integrated anatomical reference (CATCH). J Cardiovasc Magn Reson.

[5] Fukase T., Dohi T., Fujimoto S., Nishio R., Nozaki Y.O., Kudo A. (2023). Relationship between coronary high-intensity plaques on T1-weighted imaging by cardiovascular magnetic resonance and vulnerable plaque features by near-infrared spectroscopy and intravascular ultrasound: a prospective cohort study. J Cardiovasc Magn Reson.

[6] Noguchi T., Kawasaki T., Tanaka A., Yasuda S., Goto Y., Ishihara M. (2014). High-intensity signals in coronary plaques on noncontrast T1-weighted magnetic resonance imaging as a novel determinant of coronary events. J Am Coll Cardiol.

[7] Sato S., Matsumoto H., Li D., Ohya H., Mori H., Sakai K. (2021). Coronary high-intensity plaques at T1-weighted MRI in stable coronary artery disease: comparison with near-infrared spectroscopy intravascular US. Radiology.

[8] Uzu K., Kawakami R., Sawada T., Takaya T., Taniguchi Y., Hirota S. (2021). Histopathological characterization of high-intensity signals in coronary plaques on noncontrast T1-weighted magnetic resonance imaging. JACC Cardiovasc Imaging.

[9] Hajhosseiny R., Hartley A., Cole G., Munoz C., Sethi A., Al-Lamee R. (2025). Free-breathing, non-contrast, three-dimensional whole-heart coronary magnetic resonance imaging for the identification of culprit and vulnerable atherosclerotic plaque. J Cardiovasc Magn Reson.

[10] Ginami G., Neji R., Phinikaridou A., Whitaker J., Botnar R.M., Prieto C. (2018). Simultaneous bright- and black-blood whole-heart MRI for noncontrast enhanced coronary lumen and thrombus visualization. Magn Reson Med.

[11] Milotta G., Ginami G., Cruz G., Neji R., Prieto C., Botnar R.M. (2019). Simultaneous 3D whole-heart bright-blood and black blood imaging for cardiovascular anatomy and wall assessment with interleaved T2prep-IR. Magn Reson Med.

[12] Munoz C., Fotaki A., Hua A., Hajhosseiny R., Kunze K.P., Ismail T.F. (2023). Simultaneous highly efficient contrast-free lumen and vessel wall MR imaging for anatomical assessment of aortic disease. J Magn Reson Imaging.

[13] Koo T.K., Li M.Y. (2016). A guideline of selecting and reporting intraclass correlation coefficients for reliability research. J Chiropr Med.

[14] Hajhosseiny R., Hartley A., Cole G., Munoz C., Sethi A.H., Al-Lamee R. (2023). Simultaneous, free-breathing, non-contrast 3D whole-heart coronary magnetic resonance angiography and vulnerable plaque imaging in patients with suspected acute coronary syndrome. Eur Heart J.

[15] Prieto C., Doneva M., Usman M., Henningsson M., Greil G., Schaeffter T. (2015). Highly efficient respiratory motion compensated free-breathing coronary MRA using golden-step Cartesian acquisition. J Magn Reson Imaging.

[16] Henningsson M., Shome J., Bratis K., Vieira M.S., Nagel E., Botnar R.M. (2017). Diagnostic performance of image navigated coronary CMR angiography in patients with coronary artery disease. J Cardiovasc Magn Reson.

[17] Cruz G., Atkinson D., Henningsson M., Botnar R.M., Prieto C. (2017). Highly efficient nonrigid motion-corrected 3D whole-heart coronary vessel wall imaging. Magn Reson Med.

[18] Bustin A., Lima da Cruz G., Jaubert O., Lopez K., Botnar R.M., Prieto C. (2019). High-dimensionality undersampled patch-based reconstruction (HD-PROST) for accelerated multi-contrast MRI. Magn Reson Med.

[19] Nazir M., Bustin A., Hajhosseiny R., Yazdani M.F., Ryan M., Vergani V., et al. High-resolution non-contrast free-breathing coronary cardiovascular magnetic resonance angiography for detection of coronary artery disease. *J Cardiovasc Magn Reson* 2022;24 Article 26. doi:10.1186/s12968-022-00858-0. 10.1186/s12968-022-00858-0 PMC8996676 35399091

[20] Hajhosseiny R., Rashid I., Bustin A., Munoz C., Cruz G., Nazir M.S. (2021). Clinical comparison of sub-mm high-resolution non-contrast coronary CMR angiography against coronary CT angiography in patients with low-intermediate risk of coronary artery disease: a single center trial. J Cardiovasc Magn Reson.

[21] Kim W.Y., Danias P.G., Stuber M., Flamm S.D., Plein S., Nagel E. (2001). Coronary magnetic resonance angiography for the detection of coronary stenoses. N Engl J Med.

[22] Cury R.C., Leipsic J., Abbara S., Achenbach S., Berman D., Bittencourt M. (2022). CAD-RADS™ 2.0 - 2022 coronary artery disease-reporting and data system: an expert consensus document of the Society of Cardiovascular Computed Tomography (SCCT), the American College of Cardiology (ACC), the American College of Radiology (ACR), and the North America Society of Cardiovascular Imaging (NASCI). J Cardiovasc Comput Tomogr.

[23] Adamson P.D., Hunter A., Williams M.C., Shah A.S.V., McAllister D.A., Pawade T.A. (2018). Diagnostic and prognostic benefits of computed tomography coronary angiography using the 2016 National Institute for Health and Care Excellence guidance within a randomised trial. Heart.

[24] The SCOT-HEART Investigators (2018). Coronary CT angiography and 5-year risk of myocardial infarction. N Engl J Med.

[25] Halfmann M.C., Bockius S., Emrich T., Hell M., Schoepf U.J., Laux G.S. (2024). Ultrahigh-spatial-resolution photon-counting detector CT angiography of coronary artery disease for stenosis assessment. Radiology.

[26] Si-Mohamed S.A., Boccalini S., Lacombe H., Diaw A., Varasteh M., Rodesch P.-A. (2022). Coronary CT angiography with photon-counting CT: first-in-human results. Radiology.

[27] Mergen V., Eberhard M., Manka R., Euler A., Alkadhi H. (2022). First in-human quantitative plaque characterization with ultra-high resolution coronary photon-counting CT angiography. Front Cardiovasc Med.

[28] Williams M.C., Moss A.J., Dweck M., Adamson P.D., Alam S., Hunter A. (2019). Coronary artery plaque characteristics associated with adverse outcomes in the SCOT-HEART study. J Am Coll Cardiol.

[29] Doris M., Newby D.E. (2016). Coronary CT angiography as a diagnostic and prognostic tool: perspectives from the SCOT-HEART trial. Curr Cardiol Rep.

[30] Kanaya T., Noguchi T., Otsuka F., Asaumi Y., Kataoka Y., Morita Y. (2019). Optical coherence tomography-verified morphological correlates of high-intensity coronary plaques on non-contrast T1-weighted magnetic resonance imaging in patients with stable coronary artery disease. Eur Heart J Cardiovasc Imaging.

[31] Ginami G., Neji R., Rashid I., Chiribiri A., Ismail T.F., Botnar R.M. (2017). 3D whole-heart phase sensitive inversion recovery CMR for simultaneous black-blood late gadolinium enhancement and bright-blood coronary CMR angiography. J Cardiovasc Magn Reson.

[32] Wexler L., Brundage B., Crouse J., Detrano R., Fuster V., Maddahi J. (1996). Coronary artery calcification: pathophysiology, epidemiology, imaging methods, and clinical implications. Circulation.

[33] Henkelman R.M., Watts J.F., Kucharczyk W. (1991). High signal intensity in MR images of calcified brain tissue. Radiology.

[34] Suzuki S., Nishio S., Takata K., Morioka T., Fukui M. (2000). Radiation-induced brain calcification: paradoxical high signal intensity in T1-weighted MR images. Acta Neurochir (Wien).

[35] Kalisz K., Buethe J., Saboo S.S., Abbara S., Halliburton S., Rajiah P. (2016). Artifacts at cardiac CT: physics and solutions. RadioGraphics.

[36] Treibel T.A., Kelion A., Ingram T.E., Archbold R.A., Myerson S.G., Menezes L.J. (2022). United Kingdom standards for non-invasive cardiac imaging: recommendations from the Imaging Council of the British Cardiovascular Society. Heart.

[37] Truesdell A.G., Alasnag M.A., Kaul P., Rab S.T., Riley R.F., Young M.N. (2023). Intravascular imaging during percutaneous coronary intervention: JACC state-of-the-art review. J Am Coll Cardiol.

[38] Räber L., Mintz G.S., Koskinas K.C., Johnson T.W., Holm N.R., Onuma Y. (2018). Clinical use of intracoronary imaging. Part 1: guidance and optimization of coronary interventions. An expert consensus document of the European Association of Percutaneous Cardiovascular Interventions. Eur Heart J.

