# Supplementary material for: Simultaneous bright- and black-blood three-dimensional whole-heart magnetic resonance imaging for integrated coronary plaque detection and luminal stenosis assessment: A prospective comparison with coronary computed tomography angiography
Source: J Cardiovasc Magn Reson. 2026 Jan 13;28(1):102688. doi: 10.1016/j.jocmr.2026.102688 (PMC13168763; doi:10.1016/j.jocmr.2026.102688)
Supplement: Supplementary file 1 — Supplementary material [file mmc1.docx]

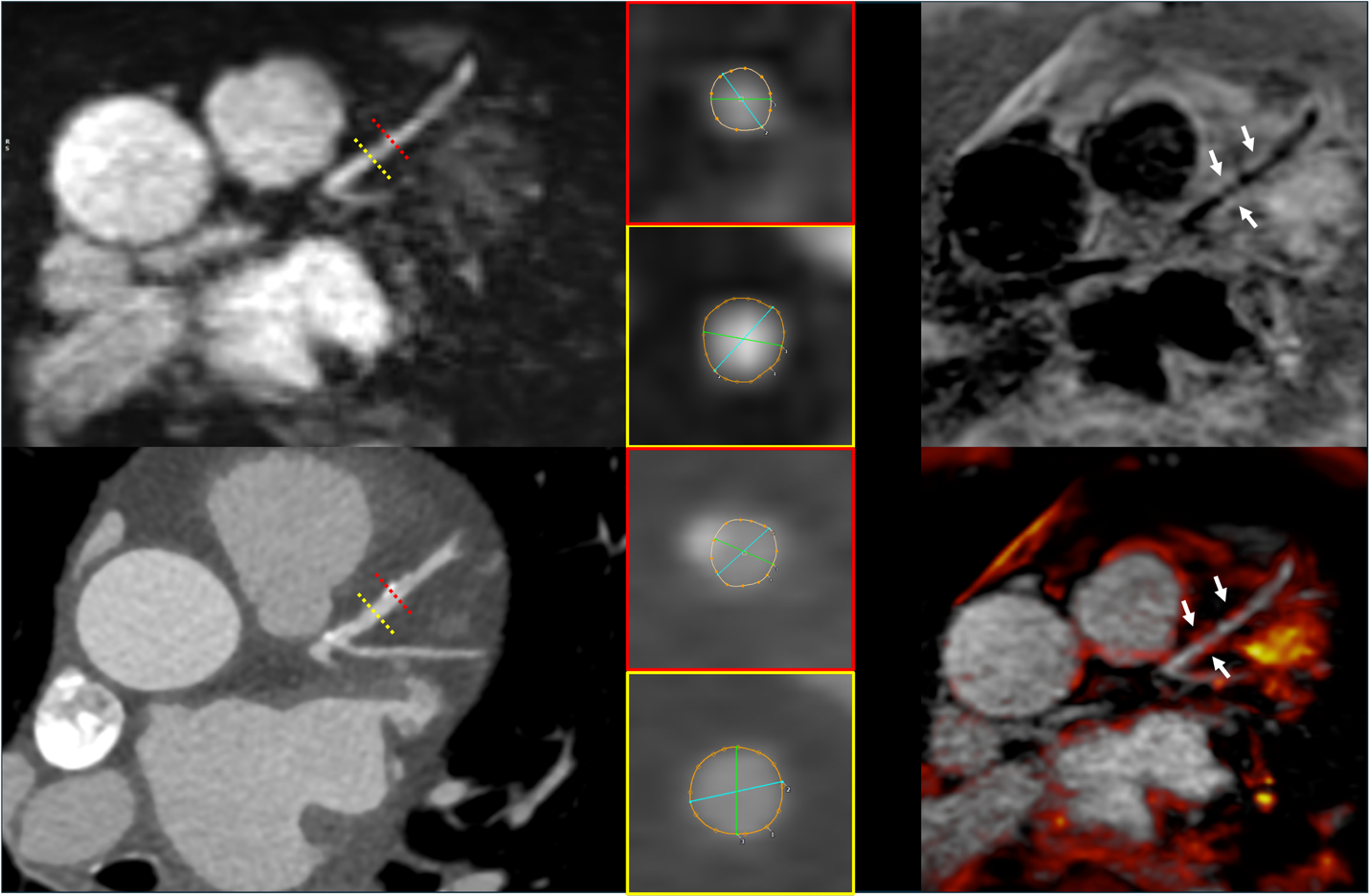


**Figure S1. Cross-sectional coronary stenosis assessment with iT2prep-BOOST and CTCA.**
Top left: iT2prep-BOOST bright-blood image; Bottom left: corresponding CTCA image. Red lines denote the location of stenosis, with corresponding cross-sectional views shown in red boxes; a region of normal vessel is indicated by the yellow line with corresponding cross sections in yellow boxes. The vessel edge is traced to determine the luminal area at the site of stenosis and the nearest section of normal vessel to calculate the percentage stenosis. Top right: BOOST black-blood image demonstrating plaque in the LAD; Bottom right: fusion image combining bright- and black-blood BOOST sequences. White arrows indicate the location of atherosclerotic plaques.

*CCTA: coronary computed tomography angiography; LAD: left anterior descending*


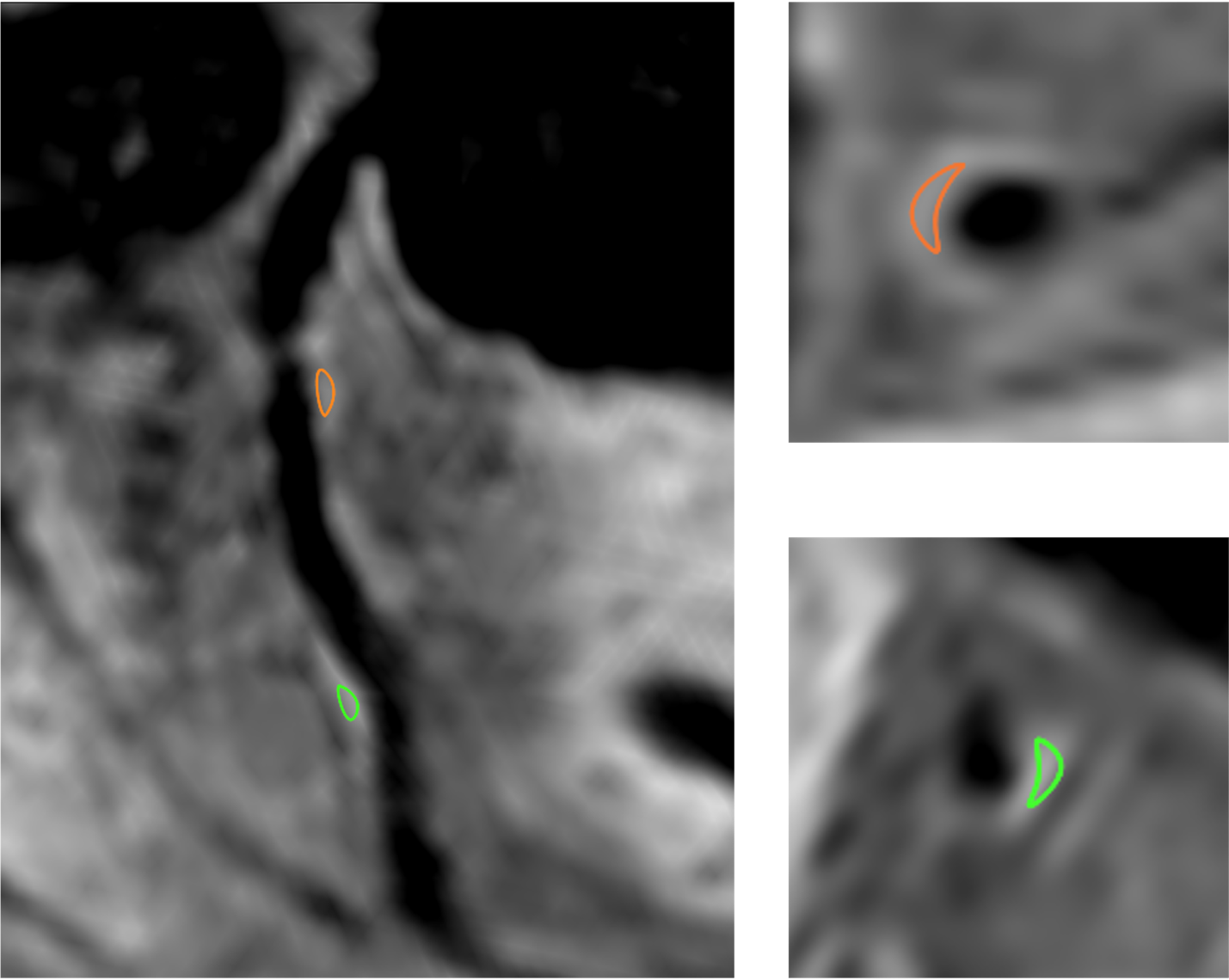


**Figure S2. iT2prep-BOOST Black-blood image demonstrating ROI placement in the coronary vessel wall.**

MPR of a coronary artery (left) show ROIs placed in areas of visually high signal within two different plaques, colour-coded orange (proximal) and green (mid). On the right, the corresponding cross-sectional images are displayed with the same colour-coded ROIs. The highest signal from any orientation is used to measure the maximal plaque signal, which is then applied for PMR calculation.

*ROI: region of interest; MPR: multiplanar reformat; PMR: plaque-to-myocardium ratio.*


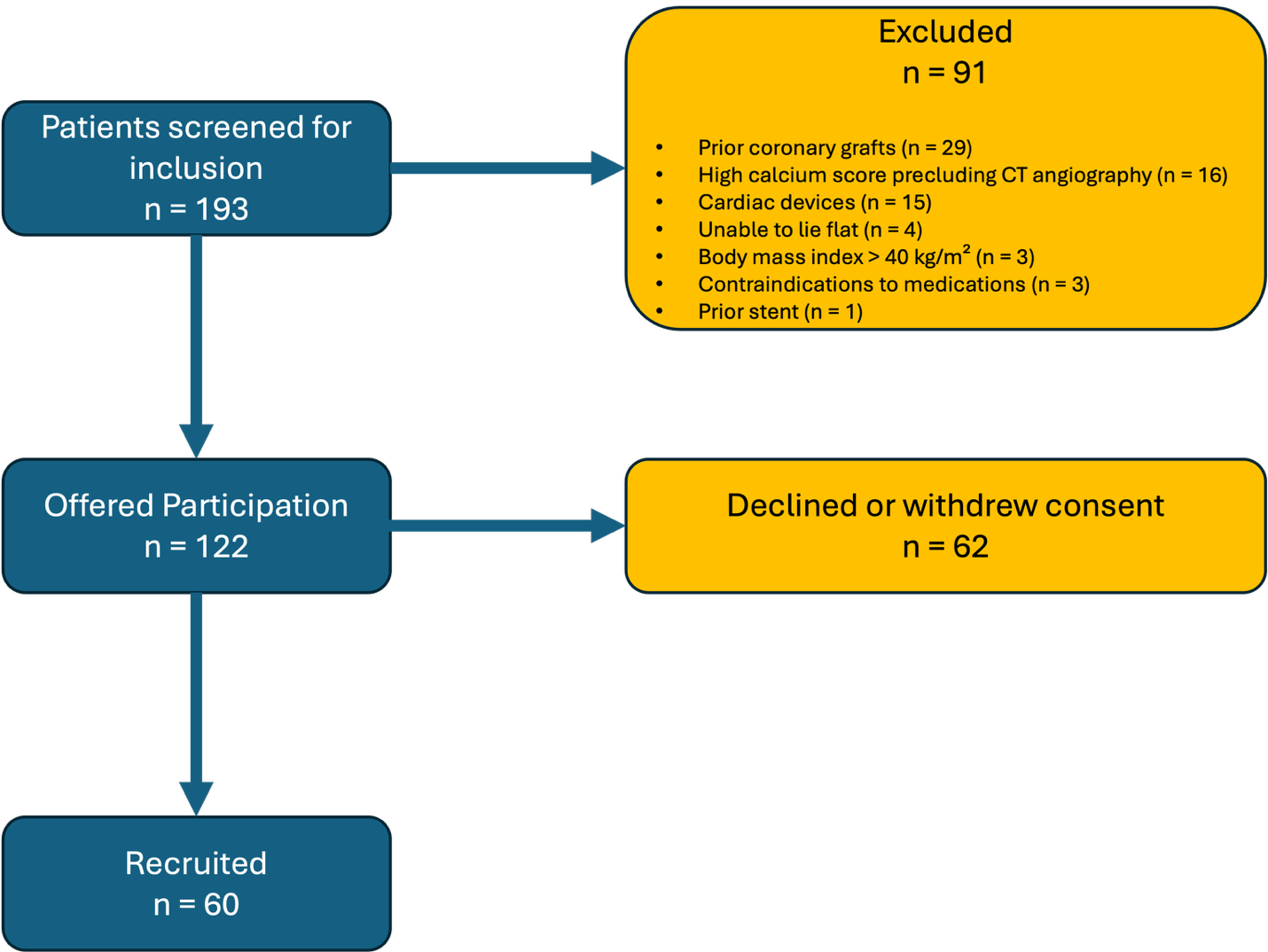


**Figure S3. Study recruitment flow diagram.**


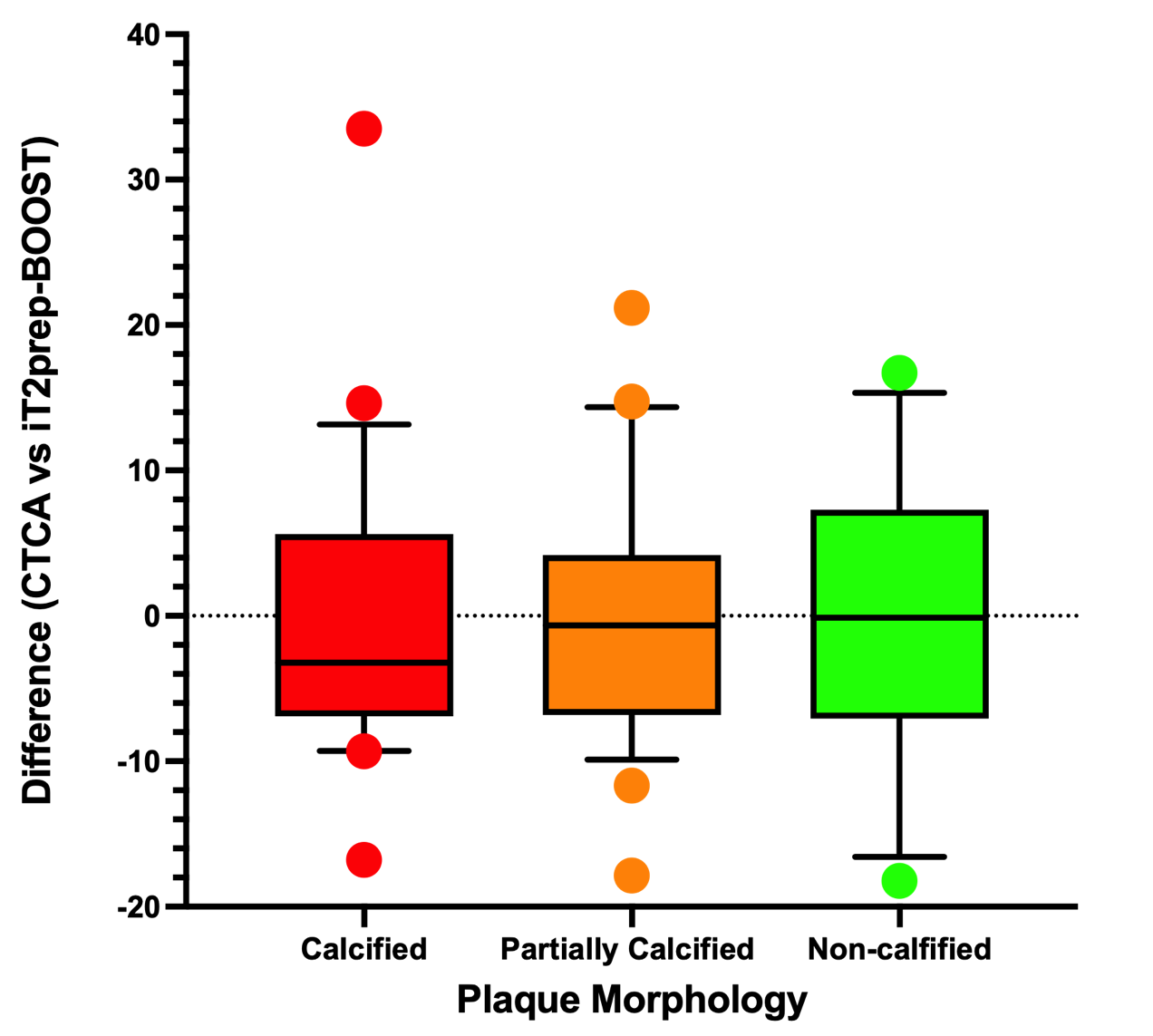


**Figure S4. Box-and-whisker plot showing the difference in percentage stenosis (CCTA – iT2prep-BOOST) across plaque subtypes.**

Boxes represent the IQR, with the line indicating the median difference. Whiskers extend to the 10th and 90th percentiles, and individual data points outside these limits are displayed. Groups include calcified plaques (red), partially calcified plaques (orange), and non-calcified plaques (green). The horizontal dashed line represents zero difference (perfect agreement). Kruskal–Wallis test indicated no statistically significant difference in stenosis bias among plaque types (p = 0.99).

*CCTA: coronary computed tomography angiography; IQR: interquartile range*


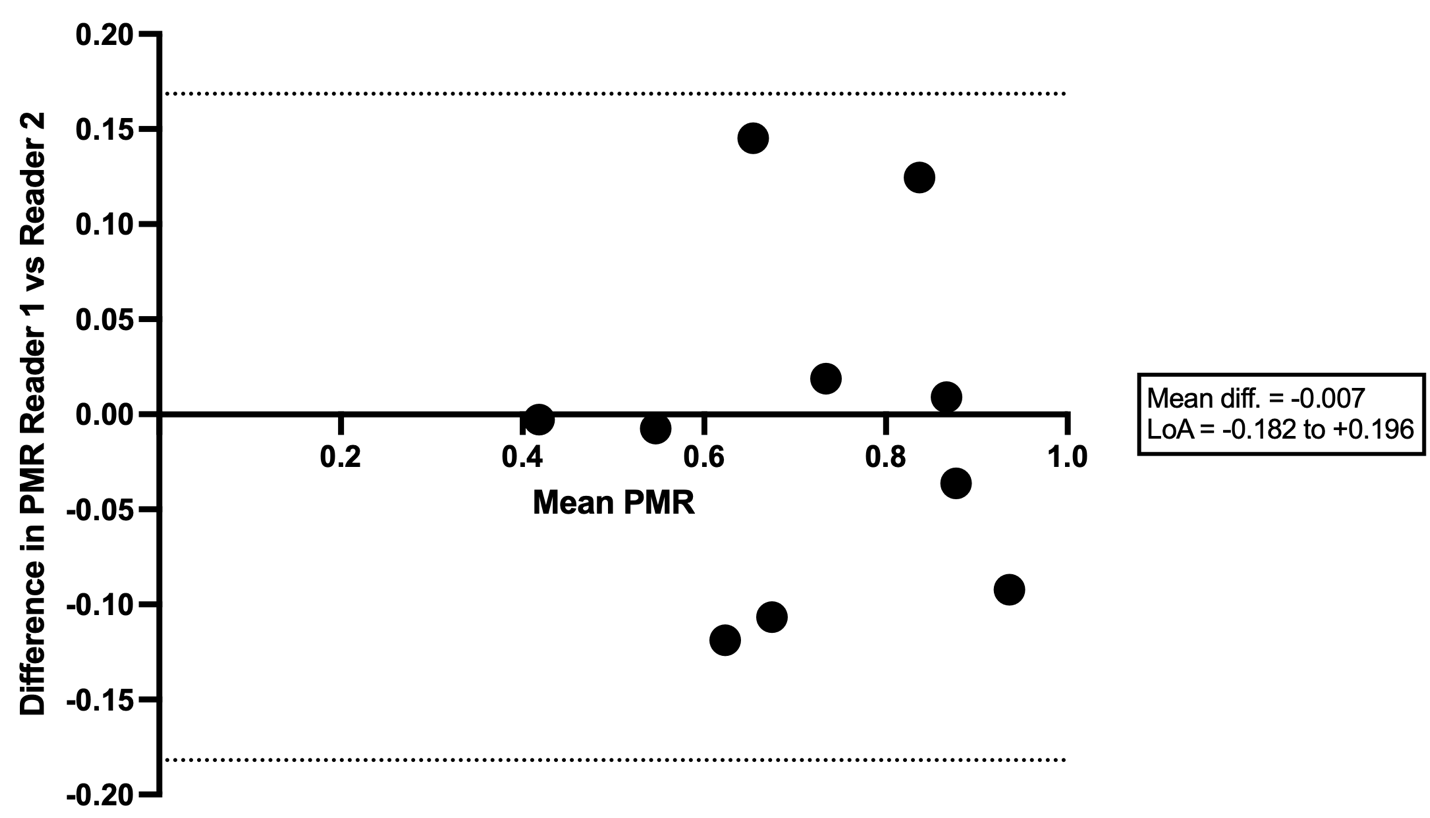
**Figure S5. Bland–Altman plot demonstrating inter-observer agreement for PMR measurements.** Each point represents the mean PMR of two independent reviewers plotted against their difference. The solid black line indicates the mean difference (−0.007), while the dashed lines represent the 95% LoA (−0.182 to +0.169).

*PMR: plaque-to-myocardium ratio; LoA: limits of agreement*
